# Acute Ingestion of a Novel Nitrate-Rich Dietary Supplement Significantly Increases Plasma Nitrate/Nitrite in Physically Active Men and Women

**DOI:** 10.3390/nu12041176

**Published:** 2020-04-22

**Authors:** Richard J. Bloomer, Matthew Butawan, Brandon Pigg, Keith R. Martin

**Affiliations:** Center for Nutraceutical and Dietary Supplement Research, School of Health Studies, University of Memphis, Memphis, TN 38152, USA

**Keywords:** nitrate, beetroot, aronia berry, red spinach, blood pressure, heart rate

## Abstract

Background: Dietary supplements purported to increase circulating nitric oxide are very popular among consumers. We determined the acute impact of two novel dietary supplements on plasma nitrate/nitrite (NOx) and nitrite alone. Methods: 20 men and women (age: 24 ± 5 years) ingested two different nitrate-rich supplements (Resync Recovery Blend at 7.5 g and 15 g; Resync Collagen Blend at 21 g), or placebo, on four different days. Fasting blood samples were obtained before and 75 min following ingestion and analyzed for NOx and nitrite. Results: Nitrite was not differently impacted by treatment (*p* > 0.05). The NOx response for men and women was very similar, with no sex interactions noted (*p* > 0.05). Condition (*p* < 0.0001), time (*p* < 0.0001), and condition x time (*p* < 0.0001) effects were noted for NOx. Values increased from baseline to post-ingestion for the Resync Recovery Blend at 7.5 g (11 ± 9 to 101 ± 48 µM) and at 15 g (9 ± 5 to 176 ± 91 µM), as well as for the Resync Collagen Blend (9 ± 9 to 46 ± 21 µM), while values for placebo remained stable (9 ± 7 to 8 ± 5 µM). Conclusion: While nitrite alone was not impacted by treatment, both Resync products result in an increase in plasma NOx, with the increase proportionate to the quantity of “nitric oxide blend” ingredients contained within each product. Future studies are needed to determine the physiological implications of the increased NOx, as pertaining to exercise performance and recovery, in addition to other aspects of human health.

## 1. Introduction

Nitric oxide is a short-lived gas that is known to promote vasodilation or “opening” of the blood vessels. Medications (e.g., Viagra^®^), foods (e.g., beets, spinach, arugula), and select dietary supplements (e.g., beetroot juice, powders, and extracts) have been used with success as agents to improve blood flow, and often give rise to nitric oxide metabolites (nitrate and nitrite), which are used as a surrogate measure of nitric oxide in blood samples. Adhering to a nitrate-rich diet is often suggested as a health-enhancing practice [1,2], in particular for those with elevated blood pressure [3].

Although mixed dietary supplements [4] and agents such as betaine [5] have been touted as nitric oxide boosters, little evidence supports these claims. Currently, the most commonly used dietary constituent to enhance nitric oxide is beetroot, which has been reported in multiple studies to increase blood nitrate and/or nitrite concentrations following acute [6] and chronic ingestion [7,8]. While results are somewhat mixed and may be due to the highly variable concentration of nitrates delivered in commercial juices [9,10], the overall consensus is that beetroot ingestion may aid certain aspects of health. The increases in nitrate/nitrite (NOx) are also often linked to improved physical performance [1,11]. In fact, many plant-based foods are known to aid vascular health, thought to be linked to the nitrate content within those plants [12,13]. 

Two products developed by the company, Resync, contain a blend of nitrate-rich foods including beetroot, red spinach extract, and aronia berry extract in addition to other ingredients that may modulate nitrate bioavailability and cardiovascular health. Resync products are sold commercially and marketed with the presumption that they can enhance blood flow and favorably impact nutrient and oxygen delivery to active muscle tissue. This may be partly due to the synergistic effect of nitrates and flavonoids [14], noting that the natural ingredients within the Resync products are rich in flavonoids. Unlike some dietary supplements that increase the nitrate content via the use of synthetic nitrate (e.g., sodium nitrate), Resync products contain plant-based forms of natural nitrate. This is an important consideration, as the natural form of nitrate has been reported to more favorably impact oxygen consumption during exercise as compared to sodium nitrate [15], highlighting the fact that other components of plants may positively affect energetics and/or nitric oxide metabolism.

Resync is marketed as an ergogenic aid and/or exercise recovery aid. The theory to support the ergogenic or recovery aspects of such products is related to the impact that enhanced blood flow may have on nutrient and oxygen delivery to active muscle tissue. If dietary ingredients can lead to improved vascular function and blood flow, such an increase in blood flow may support exercise performance and recovery. Over the past 10–15 years, multiple dietary supplements have been developed and sold based on this proposed theory. Unfortunately, very little research has supported the various claims being made [4]. 

It is thought that the combination of the plant-based ingredients (beetroot, red spinach (Amaranthus tricolor), and aronia berry extracts, in particular) will yield a significant increase in nitric oxide metabolites following acute ingestion. Therefore, the Resync products are recommended for intake 60–90 min before an athletic event and/or 30–90 min after exercise. Resync bases these claims on prior studies investigating the individual components’ abilities to increase blood NOx levels; however, there are currently no studies on the finished product itself—in particular following oral ingestion by human subjects. That said, an initial pilot experiment with a sample of two healthy subjects noted significant increases in blood NOx at 75 min following acute ingestion of Resync. The present study is the first to document these findings within larger samples of men and women. 

Based on these initial observations of increased NOx with acute Resync ingestion, coupled with the extant literature demonstrating an increase in NOx following ingestion of beetroot and red spinach, we designed the present study to determine the impact of acute ingestion of Resync on blood NOx in men and women. We hypothesized that ingestion of Resync would lead to elevated blood NOx during the post-ingestion period. Moreover, we hypothesized that the magnitude of NOx increase would be dose dependent and dependent on the concentration of nitrate-rich ingredients in each product. 

## 2. Materials and Methods 

A total of 10 men and 10 women were enrolled and completed this study. All procedures were approved by the University of Memphis Institutional Review Board for Human Subjects Research (protocol FY2020-67).

Subjects were/had:

- Aged 18–50 years,
- Male or female,
- A body mass index (BMI) between 18.0 and 29.9 kg/m^2^ (not obese),
- Non-smokers (including e-cigarettes),
- Non-vegan (due to consumption of bovine collagen),
- No diagnosed history of diabetes,
- No diagnosed history of cardiovascular disease,
- No diagnosed history of neurological disease,
- Not consumed caffeine-containing beverages within at least 48 h of testing, and
- Not pregnant, if female.

### 2.1. Initial Laboratory Visit: Screening Visit

During the initial visit to the laboratory, subjects completed the informed consent form, and health history, medication and dietary supplement usage, and physical activity questionnaires. Subjects’ heart rate and blood pressure, height, weight, waist, and hip circumference were measured. A fasting blood sample was taken and analyzed for glucose to be used as a descriptive measure and to confirm that the subject was not pre-diabetic or diabetic. To confirm non-pregnancy, females were provided with a urine pregnancy test kit, escorted to a private restroom (within the lab), and asked to perform the test. The investigators privately viewed the test results with the subject and immediately discarded the test strip into a secure trash receptacle. Subsequently, eligible subjects were scheduled for weekly testing visits after screening was completed and results confirmed. 

### 2.2. Independent Variable

Resync products currently consist of two slightly different nitric oxide blends. The Resync Recovery Blend is a dehydrated powder containing a “nitric oxide blend” of nitrate-rich foods including beetroot, red spinach extract (Amaranthus tricolor; Oxystorm^®^), aronia berry extract, turmeric root extract, ginger root extract, inulin, and hyaluronic acid, while also containing mango fruit (Careflow^TM^). The Resync Collagen Blend contains a similar “nitric oxide blend” of beetroot, red spinach extract (Oxystorm^®^), aronia berry extract, ascorbic acid, and hyaluronic acid. This collagen-based product also contains approximately 17 g of bovine collagen per serving. 

Both products were delivered to subjects in beverage form, mixed with 12 fluid ounces of bottled water. The traditional Resync Recovery Blend was evaluated after one and two servings, whereas the Resync Collagen Blend was evaluated at one serving. A placebo control condition of colored and flavored, nitrate- and polyphenol-free powder mixed in 12 fluid ounces of water was also included within the design. Therefore, a total of four different conditions were included, in random order, using a single-blind design. Each serving of powder was precisely weighed using a laboratory-grade balance prior to mixing into water. It should be noted that the label-recommended dosage of the Recovery Blend and Collagen Blend is 7.66 and 20.9 g, respectively. These amounts differ slightly from the amounts noted below that were used within the present design. When using the included scoop within each container, it was noted that the “average” powder weight was similar to what we used below.

Resync (1 serving at approximately 7.5 g, containing approximately 4.2 g of “nitric oxide blend” ingredients, as noted on the product label).

Resync (2 servings at approximately 15 g, containing approximately 8.4 g of “nitric oxide blend” ingredients, as noted on the product label).

Resync + collagen (1 serving at approximately 21 g, containing approximately 2 g of “proprietary blend” ingredients, as noted on the product label).

Placebo (7.5 g mixed in 12 fluid ounces of water).

### 2.3. Test Visit Procedures

On each test day, subjects reported to the lab in the morning hours (6–9 am) in a 10+ h fasted state and rested quietly for 20 min. Heart rate (HR) and blood pressure (systolic [SBP] and diastolic [DBP]) were measured and recorded, and a fasting blood sample was collected. Subjects then ingested the assigned condition within a 3 min period. Subjects then rested quietly for 75 min, without consuming any food or calorie-containing beverages but were allowed access to bottled water. At the conclusion of the 75 min post-ingestion period, their HR and blood pressure were measured and recorded again, and a second blood sample was collected. This concluded subjects’ participation for the day of testing. The 75 min post-ingestion time point was chosen based on prior work involving red spinach extract and the noted pharmacokinetic data [16]. 

One week later, the subjects returned to the lab for a second testing visit. During the second testing visit, subjects underwent the same procedures, with the exception of ingesting one of the three remaining conditions not taken during the first testing visit. This continued for the following two weeks, until all four conditions were completed. 

### 2.4. Blood Collection and Analysis

Single venipunctures were used to collect blood samples from subjects. Approximately 7 mL of blood was taken from subjects at each collection time. Samples were collected into Vacutainer™ tubes containing ethylenediaminetetraacetic acid (EDTA), mixed for 3–5 s, and immediately processed for plasma in a refrigerated centrifuge for 15 min at approximately 3000 rpm. The plasma was then removed from the cells following centrifugation and stored at −70 degrees Celsius until analyzed for NOx (nitrate+nitrite) using a commercially available colorimetric assay kit (Catalog#: 780001; Caymen Chemical, Ann Arbor, MI, USA). After being thawed, plasma samples were vortexed prior to being loaded into the plate. Following the addition of a nitrate reductase co-factor to each diluted sample, nitrate reductase was added, and the mixture was incubated for three hours. Greiss reagent was then added, which converts nitrite into a deep purple azo compound. The absorbance was then detected at 540 nm using a PowerWave microplate spectrophotometer (BioTek Instruments, Winooski, VT, USA) and quantification was performed using a purified external standard and construction of a calibration curve. The coefficient of variation was 4.1% for the nitrate+nitrite assay. Nitrite alone was also measured, without the addition of the enzymes, using similar procedures as described above for NOx. An investigator not involved in the dispensing of conditions and actual data collection performed the assays. 

### 2.5. Physical Activity and Dietary Intake

Subjects were instructed to follow their usual activity patterns over the course of the study period but were instructed to refrain from strenuous activity for the 24 h preceding each lab test day. Dietary intake remained similar over the entire study period although subjects refrained from ingesting nitrate-rich foods (beets, red spinach) during the day prior to each test visit. That said, water intake was not controlled for during the day prior to each test visit. In the Memphis, TN area, water quality is considered to be very good and Memphis water is known for having extremely low levels of contaminants like fluoride, nitrate, lead, and copper. Since we were interested in the impact of acute ingestion of the supplement and provided the supplement in bottled water, we do not believe that any minor differences in nitrate ingestion through water intake the day prior to testing would have impacted the values during the acute post-ingestion period. Dietary records were maintained by subjects during the day prior to each test day and analyzed using Food Processor SQL, version 9.9 (ESHA Research, Salem, OR, USA).

### 2.6. Data Analysis

The data are presented as the mean ± SD. Data were initially analyzed using a 4 (condition) × 2 (gender) × 2 (time) analysis of variance (ANOVA). No gender interactions were noted, so gender was removed from the model and the data analyzed using a 2 (condition) × 2 (time) ANOVA, with Tukey post-hoc testing and contrast analysis as appropriate. Analyses were performed using JMP software (SAS, Cary, NC, USA) and statistical significance was set at *p* ≤ 0.05.

## 3. Results

### 3.1. Overview

All 20 subjects successfully completed the study. No subject reported a problem associated with the ingestion of any condition and all conditions appeared well tolerated. Subject descriptive data are reported in Table 1. Dietary data were not different across condition days for calories (*p* = 0.77), protein (*p* = 0.82), carbohydrate (*p* = 0.98), fiber (*p* = 0.82), sugar (*p* = 0.71), or fat (*p* = 0.72). Dietary data are presented in Table 2. 

### 3.2. Heart Rate, Blood Pressure, Nitrate and Nitrite

When including gender in the model, no three-way interactions were noted for any variable (*p* > 0.05). However, as expected, gender effects were noted for HR (*p* = 0.001), SBP (*p* < 0.0001), and DBP (*p* = 0.02), with HR higher for women as compared to men and lower blood pressure for women as compared to men. 

**Figure 1:**
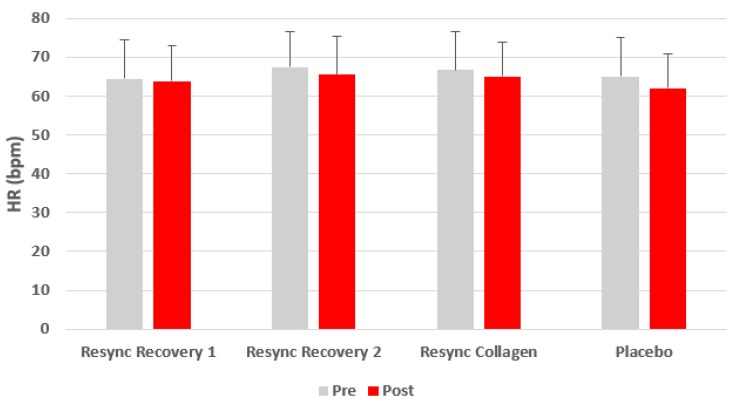
Heart rate before and 75 min after ingestion of Resync or placebo. Values are the mean ± SD. No differences of statistical significance were noted (*p* > 0.05). Note: Resync Recovery 1 = 1 serving; Resync Recovery 2 = 2 servings.

**Figure 2:**
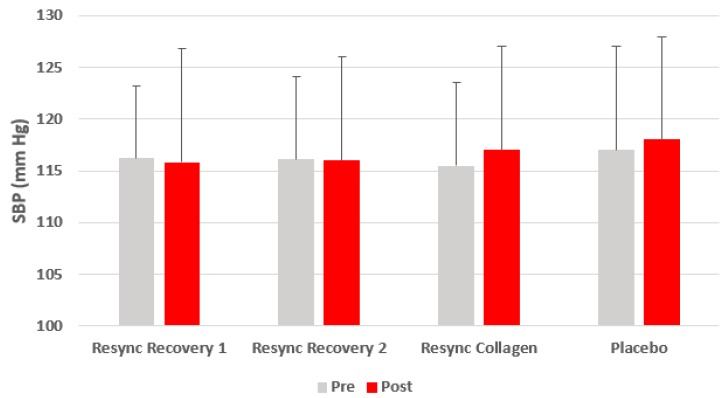
Systolic blood pressure before and 75 min after ingestion of Resync or placebo. Values are the mean ± SD. No differences of statistical significance were noted (*p* > 0.05). Note: Resync Recovery 1 = 1 serving; Resync Recovery 2 = 2 servings.

**Figure 3:**
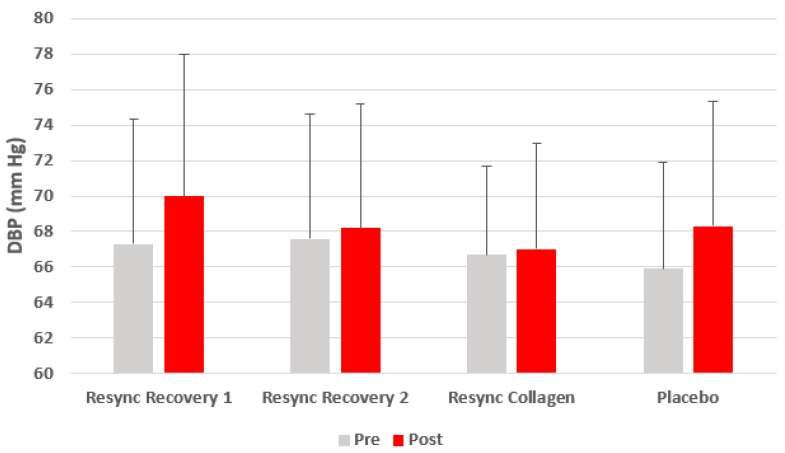
Diastolic blood pressure before and 75 min after ingestion of Resync or placebo. Values are the mean ± SD. No differences of statistical significance were noted (*p* > 0.05). Note: Resync Recovery 1 = 1 serving; Resync Recovery 2 = 2 servings.

Excluding gender from the model, no condition (*p* = 0.41), time (*p* = 0.21), or condition x time (*p* = 0.93) effects were noted for HR. No condition (*p* = 0.92), time (*p* = 0.81), or condition x time (*p* = 0.97) effects were noted for SBP. Likewise, no condition (*p* = 0.67), time (*p* = 0.17), or condition x time (*p* = 0.83) effects were noted for SBP. Data for HR and blood pressure are presented in Figure 1, Figure 2 and Figure 3. 

**Figure 4:**
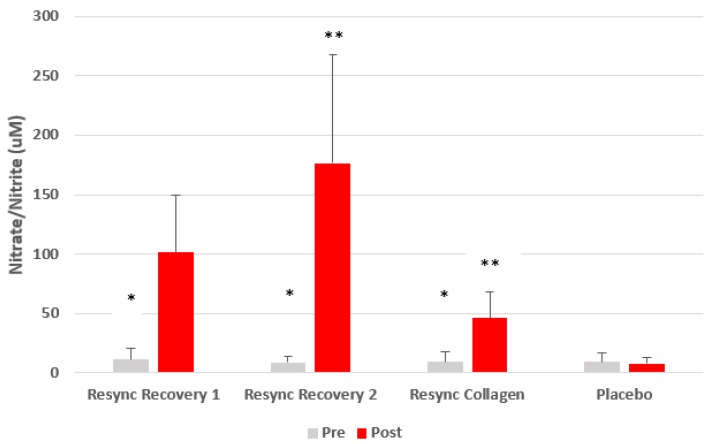
Nitrate/nitrite before and 75 min after ingestion of Resync or placebo. Values are the mean ± SD; total nitrate+nitrite. * Statistical significance from pre- to post-ingestion noted for Resync Recovery 1 (*p* < 0.0001), Resync Recovery 2 (*p* < 0.0001), and Resync Collagen (*p* = 0.002); ** post-ingestion Resync values are greater than post-ingestion placebo values (*p* < 0.05); no differences noted for pre-ingestion values between conditions (*p* > 0.05). Note: Resync Recovery 1 = 1 serving; Resync Recovery 2 = 2 servings.

Regarding NOx, condition (*p* < 0.0001), time (*p* < 0.0001), and condition x time (*p* < 0.0001) effects were noted. Regarding the condition effect, values were highest for the Resync Recovery Blend at 2 servings (92.7 µM), as compared to the Resync Recovery Blend at 1 serving (56.4 µM), the Resync Collagen Blend (27.7 µM), and placebo (8.5 µM). The change from pre- to post-ingestion was significant for both the Resync Recovery Blends (*p* < 0.0001) and the Resync Collagen Blend (*p* = 0.002), based on contrast analysis, while remaining unchanged for placebo (*p* > 0.05). Data are shown in Figure 4. 

Nitrite was minimally impacted by treatment (*p* = 0.23), with values very similar for the Resync Recovery Blend at 1 serving (2.2 µM) and 2 servings (3.0 µM), the Resync Collagen Blend (2.7 µM), and placebo (2.7 µM). It should be noted that samples for four subjects were not available for the nitrite assay, while 9 additional samples from the remaining 16 subjects were viewed as outliers (e.g., values 10-fold higher than all others) and were not included in the analysis. 

## 4. Discussion

Findings from this study indicate that the dietary supplement known as Resync significantly increases plasma levels of NOx, but not nitrite alone, following acute ingestion. This is true for the Resync Recovery Blend in a dose-dependent manner and also true for the Resync Collagen Blend, albeit at a reduced magnitude. The observed increase is significant and similar to what we have noted for sodium nitrate (unpublished findings). Interestingly, while the magnitude of increase varied across subjects for the respective conditions (see SD in Figure 4), the general pattern of increase was the same for all 20 subjects and all 20 experienced sizeable increases in blood NOx levels following each of the Resync conditions. It is possible that the combination of plant-based ingredients is targeting various pathways to increase circulating nitric oxide (e.g., reduced degradation and increased formation). Ongoing work is being conducted to determine the mechanisms of action of the Resync products. 

Over the past several years, foods thought to increase nitric oxide metabolites have been well studied and often recommended as a source of healthy nitrates [17]. Most notable is beetroot, which is delivered in various forms including juice and extract. Indeed, beetroot has been reported in several studies to have health-enhancing properties [1,18], with benefits noted in both younger and older adults [19]. Some of the health and performance benefits appear specifically related to the relatively high nitrate content of the beet, although the actual concentration of nitrates varies when considering the various commercially available beetroot juices [9]. 

Red spinach (Amaranthus tricolor) has received attention for its health-specific benefits. For example, Sani and coworkers demonstrated that red spinach has anti-cancer effects [20], while Gonzalez and colleagues recently reported that red spinach extract improved cycle time trial performance in both men and women [21]. Moore et al. [22] noted an increase in the ventilatory threshold when red spinach extract was ingested, suggesting improved physical performance capacity. Interestingly, red spinach is touted as having up to 4-fold more nitrate than beetroot extract and, based on the findings from the present study, may be chiefly responsible for the large increases in blood NOx noted in our subjects. This hypothesis is supported by the findings of Huan et al. [16] who noted an approximate 255% increase in blood nitrate levels from pre- to post-ingestion (after 65–75 min) of red spinach extract alone. Interestingly, Gallardo and Coggan (unpublished data) noted in a poster presentation that the Resync Recovery Blend had the highest per gram nitrate content as compared to five other commercially available powders. In fact, the product with the next highest concentration of nitrate per gram was less than 50% that of the Resync product. To our knowledge, the Resync product was the only one which contained red spinach extract, further highlighting the benefits of this nutrient. It is important to note that the Resync product contains ingredients with known antioxidant activity, which may help to appease concern of potential problems associated with N-Nitroso compounds due to the nitrate delivery. No evaluation of nitrate content has been performed for the Resync Collagen Blend; however, considering that it contains less of the key “nitric oxide” ingredients and resulted in a much lower nitrate/nitrite response in the present study, it is logical to assume that this product has a lower per gram nitrate content as compared to the Resync Recovery Blend. 

Aronia berry is another food that has been shown to have health-enhancing properties, likely due to the high concentration of polyphenols, anthocyanins, and flavonoids. The benefits range from reducing inflammation [23] to possessing anti-diabetic effects [24], to modulating the gut microbiota [25]. 

Aside from beetroot, red spinach, and aronia berry, the Resync Recovery Blend also contains turmeric and ginger root—both of which are also known to have potential health benefits, with a particular emphasis on modulating inflammation [26,27]. Turmeric (curcumin) has also been reported to increase plasma nitric oxide [28]. Finally, the added inulin and mango extract may support gut health [29] and microcirculation via increased eNOS activity [30]. The collective formulation may promote health benefits beyond those related specifically to nitrate; however, more extensive studies are needed to more fully evaluate the health benefits of the Resync products. 

Interestingly, we observed no alteration in blood pressure, despite the significant rise in NOx with Resync ingestion. These data agree with those of Haun and colleagues [16], who also included a sample of young and healthy men and women evaluated in a rested state. It is likely that the relatively low resting blood pressure values of our subjects were not influenced by nitrates, in the same way as might be the case for individuals with hypertension [3]. As noted in the Results section, we observed little change in nitrite itself, which has been previously reported to influence blood pressure [31]. In fact, prior research indicates that both nitrite and nitrate may alter blood pressure [31,32], as nitrate can convert back to nitrite and then to nitric oxide when physiologically required. Future investigations using Resync in conjunction with an exercise intervention are warranted to elucidate the effects of Resync on active blood pressure, in both normotensive and hypertensive individuals. Moreover, studies focused on the acute impact of Resync may need extended times of measurement for both nitrite and blood pressure (i.e., beyond 75 min) to more fully elucidate the potential impact of this supplement on these measures. 

## 5. Conclusions

The findings from this study indicate that Resync consumption results in a significant increase in plasma NOx, without differently impacting nitrite alone. The Resync Recovery Blend yields a much greater increase as compared to the Resync Collagen Blend. This is likely directly related to the quantity of “nitric oxide-stimulating” ingredients within each product. As expected, a higher dose of the Resync Recovery Blend leads to a much greater increase in the NOx concentration, which is approximately linear, in that the increase with the double dose is nearly twice that observed with the single dose. Neither supplement appears to alter resting HR or blood pressure and both are well tolerated by healthy young men and women. Future, larger-scale studies are now needed to determine the impact of repeated ingestion of the supplements, as well as the potential benefit of any NOx increase to aid exercise performance and recovery, in addition to favorably impacting other aspects of human health.

## Figures and Tables

**Table 1:** Characteristics of 20 healthy men and women.

Variable	Value
Age (years)	24 ± 5
Height (cm)	171 ± 9
Weight (kg)	71 ± 12
BMI (kg/m^2^)	24 ± 3
Waist Circumference (cm)	77 ± 7
Hip Circumference (cm)	96 ± 7
Waist/Hip Ratio	0.80 ± 0.06
Resting HR (bpm)	68 ± 9
Resting SBP (mm Hg)	115 ± 4
Resting DBP (mm Hg)	72 ± 4
Glucose (mg/dL)	80 ± 7
Anaerobic Exercise (years)	5 ± 3
Anaerobic Exercise (h/week)	3 ± 3
Aerobic Exercise (years)	6 ± 6
Aerobic Exercise (h/week)	3 ± 2

Values are the mean ± SD.

**Table 2:** Dietary data of men and women during the two days prior to each test day.

Variable	Resync Recovery Blend 1 Serving	Resync Recovery Blend 2 Servings	Resync Collagen Blend	Placebo
Kilocalories	2270 ± 913	2096 ± 563	2019 ± 642	2144 ± 896
Protein (g)	99 ± 48	93 ± 38	92 ± 34	104 ± 63
Carbohydrate (g)	241 ±104	235 ± 82	231 ± 89	230 ± 92
Fiber (g)	19 ± 10	22 ± 9	19 ± 9	21 ± 10
Sugar (g)	69 ± 46	85 ± 60	75 ± 38	72 ± 28
Fat (g)	96 ± 51	88 ± 35	80 ± 32	91 ± 55

Values are the mean ± SD. No differences of statistical significance were noted for any variable (*p* > 0.05).
